# Organization of the Influenza A Virus Genomic RNA in the Viral Replication Cycle—Structure, Interactions, and Implications for the Emergence of New Strains

**DOI:** 10.3390/pathogens9110951

**Published:** 2020-11-15

**Authors:** Julita Piasecka, Aleksandra Jarmolowicz, Elzbieta Kierzek

**Affiliations:** Institute of Bioorganic Chemistry Polish Academy of Sciences, Noskowskiego 12/14, 61-704 Poznan, Poland; julitak@ibch.poznan.pl (J.P.); ajarmolowicz@ibch.poznan.pl (A.J.)

**Keywords:** influenza A virus, RNA structure, conserved RNA motifs, RNA interactions, reassortment, packaging, vRNP

## Abstract

The influenza A virus is a human pathogen causing respiratory infections. The ability of this virus to trigger seasonal epidemics and sporadic pandemics is a result of its high genetic variability, leading to the ineffectiveness of vaccinations and current therapies. The source of this variability is the accumulation of mutations in viral genes and reassortment enabled by its segmented genome. The latter process can induce major changes and the production of new strains with pandemic potential. However, not all genetic combinations are tolerated and lead to the assembly of complete infectious virions. Reports have shown that viral RNA segments co-segregate in particular circumstances. This tendency is a consequence of the complex and selective genome packaging process, which takes place in the final stages of the viral replication cycle. It has been shown that genome packaging is governed by RNA–RNA interactions. Intersegment contacts create a network, characterized by the presence of common and strain-specific interaction sites. Recent studies have revealed certain RNA regions, and conserved secondary structure motifs within them, which may play functional roles in virion assembly. Growing knowledge on RNA structure and interactions facilitates our understanding of the appearance of new genome variants, and may allow for the prediction of potential reassortment outcomes and the emergence of new strains in the future.

## 1. Introduction

Influenza A virus (IAV) is responsible for common respiratory infection in human, spreading as seasonal epidemics and sporadic pandemics. IAV belongs to the family *Orthomyxoviridae*. Its viral genome is organized in eight, negative-sense RNA segments [1]. The viral RNA (vRNA) sizes range from ~0.9 to 2.3 kb, while the total genome size is about 13.5 kb. All vRNAs show the same organization: A central open reading frame that encodes one or more protein (in the antisense orientation) flanked by two short untranslated regions (UTRs). Segments 1–8 are named according to the encoded protein: polymerase basic protein 2 (PB2), polymerase basic protein 1 (PB1), polymerase acidic protein (PA), hemagglutinin (HA), nucleoprotein (NP), neuraminidase (NA), matrix protein (M), and nonstructural protein (NS). IAVs can be classified into antigenic subtypes, based on their surface glycoproteins: HA proteins fall into classes H1 to H18 and NA proteins fall into classes N1 to N11. Only a limited number of these HAs and NAs have been isolated from viruses known to infect humans [2,3].

The vRNA segments in the virion are organized in ribonucleoprotein complexes (vRNPs) [4,5]. Each segment, through base-pairing of the highly conserved 13 nucleotides at the 5΄ end and 12 at 3΄, forms a partially double-stranded promoter structure, which undergoes substantial structural rearrangements at certain stages of the viral replication cycle to perform distinct functions [6,7,8]. It is bound by the viral-encoded trimeric RNA-dependent RNA polymerase complex. The rest of the vRNA associates with multiple copies of NP. vRNA has been shown to fold into secondary structure motifs in both in vitro and in virio conditions [9]. Eight vRNPs (containing each of vRNA segments) are required for the production of complete infectious progeny virions. The abovementioned genome architecture provides evolutionary advantages but also complicates the process of genome packaging, which occurs in the final stage of the viral replication cycle. A major evolutionary advantage to the virus provided by genome segmentation is that it allows for genetic reassortment that is, the exchange of segments—when at least two viruses co-infect the same cell. This process is a source of genetic variability leading to the production of new viral strains with pandemic potential. However, segmentation also imposes a specific and precise mechanism of genome packaging to ensure virus propagation [10,11,12]. From two proposed models for the incorporation of eight vRNAs into infectious IAV particles, the existence of a segment-specific packaging has been repeatedly confirmed by independent researchers [12]. This process is mediated by the interaction of RNA regions called packaging signals. These regions were proposed to encompass the genome incorporation signal, which allows for packaging of that individual segment, and a genome bundling signal, which allows for the incorporation of all eight IAV segments together. The packaging sequences were initially identified in the 5΄ and 3΄ terminal coding regions of each segment [13,14,15,16,17,18]. This observation was supported by higher codon conservation in these RNA regions. Further investigation has revealed that the packaging signals are also present in central coding regions [9,19,20]. Base pairing between vRNAs in packaging regions allows for the incorporation of vRNPs into viral particles as a single supramolecular complex [21]. In virio studies have shown that low-NP RNA regions are exposed for the possible formation of RNA–RNA interactions and especially enriched in predicted secondary structure, which have been proposed to take part in the intersegment contacts [9,22]. Some reports have suggested that segments are not equally important in genome assembly; further, the process is hierarchical and has some strain-specific features [9,14,23]. Genome packaging implies limitations in terms of genetic reassortment, as the interacting vRNAs are preferentially incorporated together into the progeny virion [19]. This review collects recently published data on vRNA structure, NP association, and intersegment interactions which are important for genome assembly and have consequences for reassortment and the emergence of new strains. The presented studies have indicated key RNA-dependent mechanisms, which are known to influence the production of new genome variants of the influenza virus. The growing knowledge in this field may allow for the prediction of new strains appearing, in order to prevent infection and virus transmission in the future.

## 2. Organization of the Influenza A Virus RNA

### 2.1. RNA Structure

**Figure 1:**
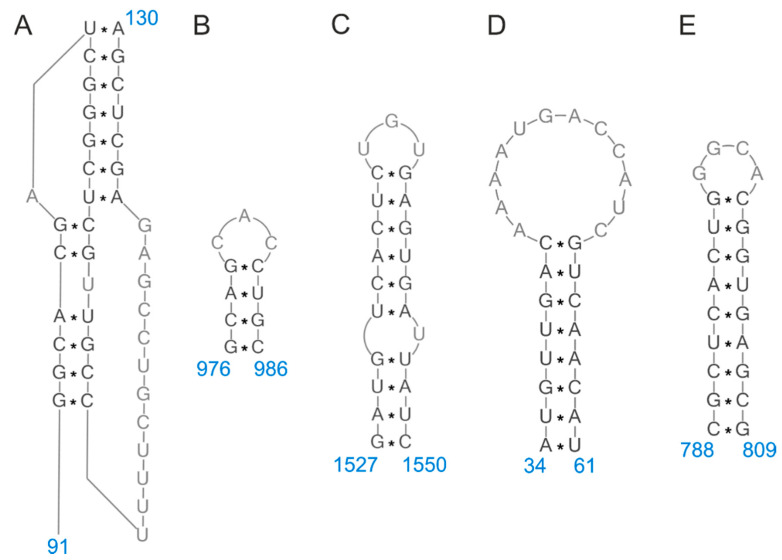
Conserved RNA secondary structure motifs in the IAV vRNA5 (**A**–**C**) and vRNA7 (**D**–**E**), confirmed by at least three independent studies and determined in virio (also included in Table 1). The structures are shown for the A/WSN/1933 (H1N1) sequence with negative sense numbering.

The correlation between the structure and function of RNA has been the subject of intensive research for years [8]. Numerous reports have shown the biological importance of the viral RNA secondary structure in key stages of the pathogen replication cycle. Influenza RNA secondary structure motifs have been thoroughly studied in vitro [24,25,26]. Based on complex experimental studies and bioinformatic analyses, structural models have been proposed for full-length naked vRNA5, 7, and 8, so far [24,25,26]. These analyses included a number of methods, such as chemical mapping, isoenergetic microarrays, RNase H cleavage in the presence of DNA oligonucleotides, evaluation of base-pairing probability, and preservation of canonical base-pairs in multiple strains. They have revealed the complex nature of vRNA secondary structures, which are organized into domains. Identified secondary structure motifs may form locally or involve long-range interactions. Besides the panhandle motif, arranged by partially base-pairing of the 5′ and 3′ vRNA terminal nucleotides, the functional role and structural dynamics of which in the influenza transcription and replication has been well-defined, other motifs have also been described [7,27,28,29,30]. Numerous hairpins, which are common in biologically relevant RNA and may perform a variety of functions, have been identified in the in vitro models [24,25,26]. The helical regions of the vRNAs are separated with bulges and single-stranded loops, which are potential sites for interactions of functional importance. The significance of the RNA secondary structure of the influenza virus has been supported by sequence-structure bioinformatic analyses showing the conservation of structural motifs, despite the high genetic variability of the virus [24,25,26,31,32,33]. Accordingly, compensatory mutations consistent with the predicted structure can be observed among strains. Mutations that occur in conservative regions usually allow for the maintenance of canonical base pairs. Significant conserved RNA structural motifs with potential functional roles are described below and summarized in Table 1. Consensus vRNA5 structure prediction for a number of strains belonging to different subtypes has revealed common structural motifs in regions 1436–1475, 1476–1530 (Figure 1A), 922–938, 577–593 (Figure 1B), 89–105, and 16–39 (Figure 1C) (positive sense numbering) [33]. Hairpins 16–39 and 578–592 (1527–1550 and 974–988 in negative sense numbering, respectively) have also been identified in in vitro structural analyses of vRNA5 as conserved among influenza type A strains [25]. A number of highly conserved hairpins have also been detected in vRNA7 regions: 34–61 (Figure 1D), 144–166, 337–357, 518–550, 695–710, 721–737, 762–786, 788–809 (Figure 1E), and 828–846 [26]. Studies of vRNA8 have revealed five stem regions spanning nucleotides 261–270/277-288, 312-317/322-327, 696-701/775-780, 704-713/758-767 and 736-740/744–748, which are predicted to form across all analyzed strains (over 14,000 sequences) [24]. Structural analysis within the packaging signals revealed the presence of conserved structural motifs in vRNA5, 7, and 8 [24,25,26]. Four out of six predicted consensus vRNA5 motifs (16–39, 89–105, 1436–1475, and 1476–1530) were located within defined packaging signals [33,34]. The highly conserved hairpins 87–115 and 1483–1497 were found in previously determined 5′ and 3′ packaging regions of vRNA5 [19,25]. A similar role in vRNA7 has been suggested for the highly conserved hairpin 34–61, as well as motifs formed in regions 1–182 and 687–875 of vRNA8 [26,35,36,37,38,39]. Structure analyses carried out at various temperatures allowed for the identification of motifs or RNA regions with high thermodynamic stability, as well as those that are more dynamic and may undergo rearrangements [25,26]. As an example, consider vRNA7 of A/Vietnam/1203/2004 (H5N1) (Vietnam), region 965–1007 at the 3′-end, which adapts different conformations depending on the temperature [26]. Interestingly, this region overlaps with a previously detected packaging signal spanning nucleotides 966–983 [16]. Structural dynamics may be important for RNA function; however, this remains to be directly proved. Structural changes may also be vital for virus adaptation to new hosts and further investigation may facilitate the prediction of strain evolution. In vitro studies face certain limitations, such as a lack of interactions with proteins and other factors that may affect the final spatial organization of RNA. However, the significance of conserved structural motifs has been confirmed through the introduction of mutations [33]. The in vitro determined conserved secondary structures have also been targeted by antisense oligonucleotides [25,37,40]. The obtained inhibition of influenza virus replication supports the structure and functional role of secondary motifs in the viral life cycle.

Results of mapping experiments performed in cellulo, in vivo, and ex virio—conditions in which interactions with proteins and other factors occur—are often difficult to interpret. The availability of nucleotides may be affected by the existence of additional interactions, rather than their involvement in the formation of secondary structure motifs. Although RNA structure mapping has been conducted in vivo in several studies, their conclusions were modest and careful, focusing on thermodynamically stable, small local motifs. This is reasonable when interpretation is hard, due to the many factors that could influence chemical mapping results. These factors include RNA–RNA interactions, such as with viral RNA and with proteins (e.g., viral proteins). The other difficulty are the limitations of RNA folding programs, which expand with the length of the studied RNA. Incorporation of experimental data can greatly facilitate RNA structure prediction; however, not all of the RNA folding rules are known and, even though many are known, some of the defined rules are chosen by different prediction programs, in order not to be incorporated in parallel during calculation, due to the complexity of such folding. At present, researchers may routinely choose several individual options: prediction without pseudoknot, pseudoknot prediction separately, choosing pairing distance (usually 600 nt, but also 150 nt), or no pairing distance. These options generate different secondary structures, which all need to be considered and coupled with other experiments.

Recently, a work has been published on the vRNA structure in purified A/WSN/1933 (H1N1) (WSN) virions (in virio) and in vitro transcribed RNA segments or deproteinized virus particles (ex virio), as analyzed by selective 2′-hydroxyl acylation analyzed by primer extension and mutational profiling (SHAPE-MaP) coupled with next-generation sequencing (NGS) [9]. This study confirmed that each genomic segment adopts a unique RNA organization in virio. Regions of low SHAPE-MaP reactivity indicate base-pairing of the nucleotides and RNA secondary structure formation. The 5′ end of each segment is especially enriched in structural motifs, mainly hairpins with high-probability of base-pairing in the stem regions and short helices forming locally. Base-pairing of the terminal 5′ and 3′ nucleotides in each segment was confirmed. The work focused on determining locally constrained RNA structures, where the maximal pairing distance was established as less than 150 nucleotides. Therefore, long-range secondary motifs—except panhandle—were not identified. These results demonstrated that some of the RNA motifs can be found in both in virio and in vitro conditions. A subset were also consistent with previous in vitro structural studies of the Vietnam strain. Hairpins in vRNA5 regions 406–422, 460–476, 976–986 (Figure 1B), 1363–1375, 1484–1496 and 1527–1550 (Figure 1C) have been identified in both in virio WSN and in vitro Vietnam strains [9,25]. A number of recurrent hairpin motifs are also present in vRNA7 regions 34–61 (Figure 1D), 138–171, 337–357, 578–585, 695–710, 768–779, and 788–809 (Figure 1E) [26]. Interestingly, the hairpin regions 34–61 and 788–809 have been previously described as packaging signals important for virion assembly [16,35,36,39,44]. Common motifs for the two abovementioned strains have also been found in vRNA8 regions 261–288, 293–298/342–347, and 312–327 [9,24]. The data support secondary structure conservation and suggest its functional role. Experiments performed on different IAV strains—WSN, A/Puerto Rico/8/34 (H1N1) (PR8), and A/Udorn/72 (H3N2) (Udorn)—showed that RNA with high sequence identity have similar conformation [9]. Additionally, the authors concluded that the presence of NP makes RNA less structured.

### 2.2. NP—RNA Association

It has been found that NP binds to vRNA in a non-uniform manner [41,45]. This feature allows for RNA secondary structures to form in low-NP regions. However, these studies could not exclude that NP binding might also be affected by local secondary structures. In one of the published reports, the NP–RNA association was investigated during PR8 infection in 293T cells by photoactivatable ribonucleoside enhanced cross-linking and immunoprecipitation (PAR-CLIP) coupled with NGS [41]. On average, NP is associated with 12 RNA nucleotides. The results showed that, for this particular strain, low-NP-binding regions encompass about 10% of the viral genome. These regions are especially enriched in stable RNA secondary structures characterized by the presence of hairpins, as bioinformatically predicted by the authors [41]. Among these motifs, a pseudoknot in vRNA5 region 1410–1495 (Figure 1A) was predicted, the role of which is further described in the RNA interactions section. RNA motifs have been hypothesized to interact with either each other or host and virus factors. Many of them are located in the terminal regions of segments within previously predicted packaging signals.

Another study defined the binding profile of NP for each IAV vRNA segment in the infected cell culture supernatant using high-throughput sequencing of RNA isolated by crosslinking immunoprecipitation (HITS-CLIP) coupled with NGS [45]. The NP–RNA association for all eight vRNA segments of WSN and A/California/07/2009 (H1N1) (California) has been mapped. The HITS-CLIP profile between replicates was consistent. The conducted experiments allowed the authors to conclude that NP binding profiles to the vRNA of both H1N1 strains were non-uniform in virio. NP was not regularly spaced across vRNAs and was almost absent in extended regions of several segments, such as PA and HA segments. Despite the fact that both IAV strains were of the same subtype (H1N1), there were similarities in their NP binding profile, but also explicit disparities in the association NP with vRNA. NP binding to PA and HA segments was moderately correlated for the tested strains, while the correlation for PB1 and NS segments was poor. Furthermore, RNA-binding domains of the two NP protein variants did not account for the difference in NP-vRNA binding profiles observed between strains. Based on the conducted research, a vRNA structure model has been proposed, in which certain vRNA regions are strongly associated with NP, while others may dynamically associate and disassociate from NP. Even similar H1N1 IAV strains have individual non-peak regions, while their overall NP binding profile is congruent. It has also been suggested that bias in nucleotide composition may be an important determinant of NP binding. Compared to the overall IAV genome, NP molecules preferentially associate with G-rich and U-poor vRNA regions.

Recent reports have also shown that vRNPs may present heterogeneous conformation and dynamic structure [46]. In cryo-EM studies concerning WSN, it has been observed that NPs can adopt a wide range of positions in RNA strands. Moreover, vRNA has been defined as the most flexible component of the complex. vRNP interstrand interactions are dynamic. The use of nucleozin—an antiviral compound which induces the formation of NP aggregates—indicated the role of NP in influenza replication cycle. The inhibitor caused transcription elongation blockage. Further analysis of EM images revealed that vRNP maintained compact helical structures during transcription. A mechanism called “processive helical track” has been proposed, in which a sliding movement between two RNA strands facilitates polymerase movement along vRNA. In this process, the polymerase remains bound to both ends of the vRNA at all times, except for the step in which the 3′ end is amplified. The template during amplification is temporarily detached from NP. However, the postulated roles of NP are extensive and contradictory, depending on the cycle stage: From maintaining the helical structure and stabilizing vRNP to melting the vRNA structure and sustaining its accessibility during amplification. NP takes part in interstrand contact, which has to be remodeled during transcription. Nucleozin caused local conformational changes in vRNP, which propagated gradually across the whole helix, where no replication or transcription occurred. A similar process can also take place during replication, as it has been shown previously that nucleozin may also inhibit the cycle at this stage. The above published data are complementary to the RNA–RNA interaction findings discussed below.

## 3. RNA Interactions

Only a complete virion, containing exactly one copy of each RNA segment, is able to infect host cells. Selective packaging of segments is a topic which has been thoroughly investigated by researchers. Electron microscopy images have provided information on the overall genome architecture, where the seven vRNPs are arranged around a central one, regardless of the tested strain [47,48,49,50]. This architecture seems to be vital for the virion, as it is maintained even in extreme situations such as when only seven vRNAs, are present [51]. It has been found that the segmented IAV genome is assembled and packed in viral particles by the action of coordinated packaging sequences and specific amino acids in NP. It has also been previously shown that the packaging of genome segments is strictly dependent on sequences close to the 5′ and 3′ termini [15,17,36,38,52,53]. The experiments were performed involving constructs with vRNA terminal sequences and reporter genes in the coding regions. The results indicated that these mutated segments may be incorporated into progeny virions and allow for the expression of reporter genes. Genetic engineering of IAV and IBV has shown that certain manipulations of terminal sequences allow for reassortment between the virus types, which never takes place in nature [54]. This is also consistent with the occurrence of defective interfering RNAs, which are vRNA segments characterized by large deletions in the open reading frames but preserving original terminal sequences and the ability to assemble into nascent particles [21]. Apart from that, internal coding regions have also been found to engage in the packaging process [19,38,41,55].

The mutagenesis of regions or residues selected as candidates for functional packaging signals and successive investigations into their effects on viral production provide a valuable source of information. The role of packaging signals within coding regions has been investigated through the introduction of synonymous mutations, which altered genome assembly [36,43]. The sequences were found by analysis of reduced synonymous codon variation in sets of known influenza sequences. The results showed that specific residues play significant roles in vRNP arrangement, ensuring efficient genome packaging. Mutagenesis-based studies have indicated that changes in a single segment can disturb assembly and cause a decrease in the efficiency of packaging of other segments. Experimental data have shown that RNA–RNA interactions between segments are important for the process. In virio chemical mapping results support the accessibility of exposed RNA regions for intersegment interactions [9]. The functional role of a direct interaction between two vRNAs was first demonstrated for vRNA2 (nucleotides 289–309 in positive sense numbering) and vRNA8 (257–277) by electrophoretic mobility shift assay [19]. Structure modeling has suggested that the abovementioned regions fold into hairpins, which may participate in the RNA–RNA contacts through kissing-loop interactions. The role of loop interaction has been confirmed through the application of antisense oligonucleotides targeting the segment interface, which reduced vRNA–vRNA binding. Mutagenesis confirmed the roles of these regions in virion packaging. Synonymous mutations have also been used in a study investigating in silico predicted hairpin structures in vRNA7, which are highly conserved among influenza strains and located within the packaging signals [16,35,39]. These experiments confirmed that structure-disturbing mutations in coding regions 219–240 and 967–994 (positive sense numbering) may disrupt replication and increase the production of defective virus particles, suggesting that structural motifs mediate genome assembly and are involved in packaging. Region 967–994 has also been predicted as low-NP binding in a report analyzing NP–RNA association patterns [45]. The structural significance of hairpins 219–240 and 967–994 has been supported by other studies, which also indicated hairpin formation both in vitro and in virio (Figure 1D,E) [9,26,43,44].

Research on packaging signals has also been performed using viruses carrying premature termination codons in HA or M2 (matrix protein 2) and containing random sequences (10–12 nucleotides) in the 3′ bundling signals of these segments [43]. These experiments were performed in cells expressing complete viral HA or M2 proteins in trans. The presence of premature termination codons did not affect genome packaging when intact proteins were expressed by the cells. Introduction of a random sequence in bundling signals affected the viral replication cycle in various ways. Viruses which were able to propagate despite changes were selected to identify residues required for proper genome packaging. The conducted experiments included the chemical mapping of vRNP and structural analyses. The obtained results led to the conclusion that single mutations in selected codons may induce changes in local vRNP structures but do not significantly affect RNA–RNA interactions. However, substantial alterations or their synergistic action may cause global structure changes and the disruption of multiple RNA–RNA contacts, with serious consequences on genome packaging [43].

Similar conclusions concerning packaging sequences have been drawn in experiments conducted on the A/SC35M H7N7 (SC35M) influenza strain [22]. To compare single and multiple packaging sequences effect on the viral genome packaging, up to eight synonymous codons were inserted into the conserved 5′ terminal coding region of vRNA1 nucleotides 2245–2268, vRNA2 2257–2280, and vRNA3 2137–2160 (positive sense numbering). These mutation sets were selected due to being previously described as causing specific genome packaging defects when individually introduced into vRNA segments of the WSN strain [18]. Through these experiments, it was demonstrated that, while the vRNA2 mutant produced an increased number of non-infectious viral particles, the genome packaging of the other two vRNA mutants was not impaired [22]. Distinct genome packaging defects were caused by combinations of the mutated packaging sequences in different vRNA segments. The comparison of results obtained from SC35M and WSN experiments revealed that the effect of a single mutated packaging sequence is strain-dependent. In SC35M, single mutations in packaging sequences in three diverse vRNA segments were tolerated by the vRNP interaction network, but their simultaneous combination caused packaging defects, described by the absence of distinct vRNP subsets from the virions. It was speculated that this resistance to mutation of the vRNP network is linked to the involvement of many possible intersegment RNA–RNA interactions and the network plasticity.

Mutational studies of vRNA5 terminal packaging regions defined as low-NP binding in PAR-CLIP analysis have been carried out, in order to prove the RNA structure-function correlation [41]. These regions, covering nucleotides 22–68 and 1410–1495 (positive sense numbering), were bioinformatically predicted to form a hairpin and pseudoknot (Figure 1A), respectively. The pseudoknot in region 1410–1495 has also been proposed in previous studies [33]. In the in vitro structural studies, this region (87–115 in negative strand numbering) was predicted to form a hairpin, which was successfully targeted by an antisense oligonucleotide, causing substantial viral inhibition in the cell culture [25]. The in virio structural analysis also proposed a pseudoknot (nucleotides 79–134); however, the nucleotide reactivity profiles did not exclude the presence of a hairpin [9]. The roles of selected motifs in the progeny virion assembly have been hypothesized [41]. The results showed that synonymous mutations destabilizing the expected RNA structure have inhibitory properties for viral replication. Mutations which do not disturb the RNA structure in these regions allow for maintenance of virus proliferation at the wild-type level. Conversely, structure-disturbing nucleotide changes in the intermediate NP-bound regions (vRNA5 nucleotides 145–175, 456–490, 584–608, and 1058–1081) do not affect virus replication. The abovementioned results have been confirmed both in an MDCK cell culture and in mice. Structure disruption in the critical motifs (vRNA5 nucleotides 22–68 and 1410–1495) was also associated with an increase in defective viral particle occurrence. Investigation of progeny viral particles showed changes in the segment stoichiometry, indicating packaging defects. Similar experiments have been carried on other segments. Synonymous structural mutations in vRNA1 low-NP region 1823–1944, vRNA2 497–561, and vRNA8 22–86 resulted in viral attenuation in infected MDCK cells [41]. The production of mutant viruses was also decreased in mice. Mutations preserving and disrupting RNA structure in NP-abundant regions (vRNA1 350–375 and 2213–2239, vRNA2 1828–1858 and 2032–2058) did not influence virus titer. Mutation in the vRNA8 22-86 region caused defective packaging of vRNA3, 4, 5, and 7. Mutation in vRNA2 497–561 reduced the packaging of vRNA6, while vRNA1 1823–1944 mutation did not change the packaging. The quoted results support the roles that RNA secondary structure motifs play in packaging, which are present mainly in regions of non-abundant NP–RNA binding. Next, mutations in vRNA1 1823–1944 and vRNA5 1410–1495 were also introduced to two other strains–A/shorebird/Delaware/22/2006 (H7N3) and pandemic California—in order to check their effect on virus proliferation. The results obtained for strains were consistent—structure-affecting mutations lead to decreased viral replication and defective genome packaging. These results led to the conclusion that conserved RNA motifs important to the viral replication cycle are preserved among related strains. The roles of high-NP vRNA regions were discussed and the authors suggested that they could participate in NP-based interactions between segments. The organization of RNA may be adapted to improve protein binding.

Recently, RNA–RNA interactions have been investigated in virio in WSN using the sequencing of psoralen-crosslinked, ligated, and selected hybrids (SPLASH) method and validated by qPCR [9]. A complex network of interactions between RNA segments was detected. The identified interaction loci can possibly establish a range of RNA–RNA contacts with a subset of more prevalent interactions. The experimental data suggested some degree of plasticity in these interactions, as well as RNA structural dynamics allowing a single region to interact with multiple segments. The structure formed by the specific interaction loci was determined, with respect to SHAPE-MaP reactivity constraints. The calculated free energies (ΔG) of predicted intersegment interactions were highly favorable. Moreover, the interaction loci tend to be more structured than other vRNA regions, according to nucleotide reactivity analyses. This suggests that RNA motifs participate in the interactions and influence them. A comparison of the SPLASH results obtained for two related strains (96% sequence identity)—WSN and PR8—revealed the common core of the interactions. PR8 and WSN shared a part of the top interaction loci. Some of the top PR8 interaction loci were present in WSN, as minor interactions. There were also unique, strain-specific interactions. A similar comparison has also been carried out for the more distant Udorn strain, which revealed some common interactions; however, more differences in the RNA–RNA contacts were reported [9].

Multiple intersegment interactions of WSN concentrated around hotspots have also been detected in virio in a study using dual crosslinking, immunoprecipitation, and proximity ligation (2CIMPL) [20]. About 10% of the detected interactions overlapped with those identified by the SPLASH method. The most predominant interaction loci was found in the central region of vRNA5, adjacent to a strong NP protein binding site; showing that this kind of region may also participate in RNA–RNA interactions. Some of the identified interactions were detected within NP-abundant regions, suggesting that protein binding does not exclude RNA-RNA contact. The vRNA5 hotspot, particularly region 656-705, was mutated using synonymous codons, in order to elucidate its role in virion production through reverse genetics [20]. These changes did not affect significantly viral replication; however, 2CIMPL analysis showed that the mutations caused interaction rearrangements, loss of the vRNA5 hotspot, and the formation of new prevalent interaction loci in vRNA3, 4, and 6, thus creating more thermodynamically favorable duplexes.

Recently, a comprehensive study of RNA-RNA interactions using high-throughput sequencing techniques has been reported [42]. Crosslinking, ligation, and sequencing of hybrids (CLASH), ligation of interacting RNA followed by next generation sequencing (LIGR-seq), dimethyl sulfate and next generation sequencing (DMS-seq), and selective 2′-hydroxyl acylation analyzed by primer extension and next generation sequencing (SHAPE-seq) were performed in virio. A region of low reactivity spanning nucleotides 87-130 in vRNA5 of PR8 was detected and confirmed by two methods (SHAPE-seq and DMS-seq). The reactivity of this region was not changed in vRNA, vRNP, and virion, suggesting secondary structure conservation across these forms. Interestingly, the region spanning nucleotides 1–100 has been defined as being engaged in the intersegment interactions with vRNA3, 6, and 8. In previous studies, the abovementioned region was localized in the low-NP binding RNA region and predicted to form a pseudoknot (Figure 1A) [33,41,43,44]. Again, mutations designed to disrupt this structure caused impaired propagation of the virus, replication and packaging of vRNA3, and segment bundling, in agreement with previous studies [42]. Segment interaction rearrangement was also observed. In the mutant virus, the intersegment contacts between region 1-100 in vRNA5 and vRNA3 were abolished, as well as other interactions of vRNA3 with vRNA2 and vRNA6 identified in the wild-type virus. Additionally, a novel interaction locus was formed between vRNA3 and vRNA2. These results were consistent with other reports postulating the redundancy of RNA-RNA interactions in the influenza virion.

These recent findings were also compliant with previous reports concerning human A/Moscow/10/99 (H3N2) (Moscow), avian A/Finch/England/2051/91 (H5N2) (England), and WSN strains [21,49,56]. The results showed that interaction networks and sequences differ between strains. Some degree of intrastrain differences were also considered acceptable. Moscow budding virions were visualized by electron tomography, which revealed that the arrangement of the four longest segments is conserved for this strain [21]. An in vitro electrophoretic mobility shift assay indicated that all genomic segments were engaged in a single interaction network. Each vRNA formed at least one interaction with other vRNA. The author suggested that the interaction sites are probably located in protein-free regions; however, NP may support intersegment contacts. New findings have shown, however, that NP-binding does not exclude the formation of RNA-RNA intersegment contacts [20]. The overall organization of the genome is a supramolecular complex, in which the central vRNP is surrounded by seven vRNPs held together by vRNA-vRNA and NP-NP protein contacts [21]. The identified vRNA-vRNA interactions were also relevant packaging signals in WSN and PR8 strains [48]. A similar single network of interactions has also been determined in in vitro studies carried out on avian England strain [56]. However, the network and interacting regions were found to be distinct from those previously defined for Moscow and PR8. In this case, segment interactions were more distinguishable in the central coding regions, rather than in terminal regions. Numerous contacts between vRNPs were detected in electron tomography and their overall distribution all along vRNAs was consistent with the in vitro observations [56].

## 4. Implications for the Emergence of New Strains

The segmented genome of the influenza virus is a source of genetic diversity, obtained through a process of antigenic shift. This phenomenon is responsible for significant antigenic changes. The reassortment of segments between animal and human strains may lead to the production of strains with pandemic potential. Certain exchange and composition of segments enables transmission from animals to humans, sustained person-to-person transmission, and other features carried by new strains [57]. Co-circulation of strains in the environment and the acquisition of adaptive mutations poses a serious medical threat for the human population. Besides, reassortment also contributes to the worldwide spread of H3N2 strains resistant to adamantine, which cause severe seasonal epidemics (e.g., the Fujian-like outbreak in 2003/2004) [58,59]. Furthermore, oseltamivir-resistant influenza variants have been distributed by reassortment [60]. It has been shown that segments tend to co-segregate during the packaging of progeny virions; this feature may be an important constraint affecting vRNA exchange. Experimental efforts have aimed to predict the potential for reassortment between strains. It is known that some genetic constellations, which are genetically and functionally stable, allow for the maintenance or even enhancement of viral replication; these may serve as the source of new, highly pathogenic strains. Research goes on to indicate key features of the particular segments, which allow or block reassortment. These analyses may improve disease surveillance and help to control its spread.

**Figure 2:**
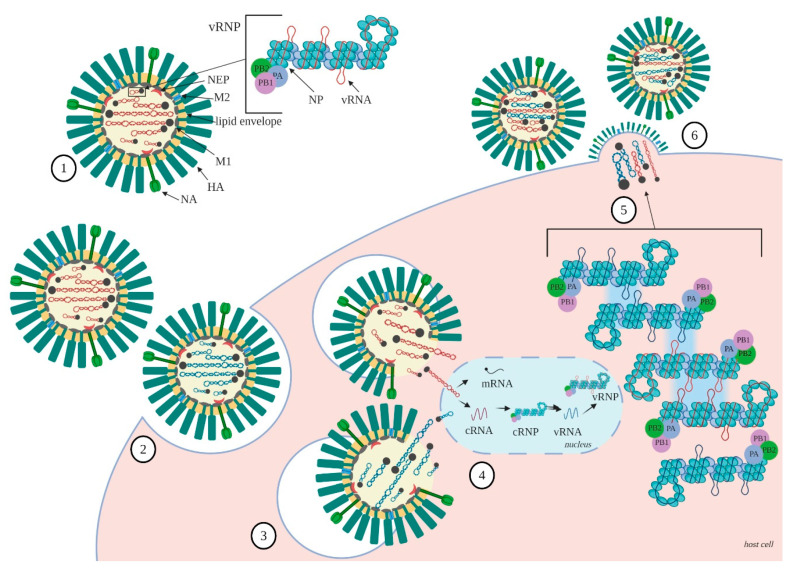
Influenza virus replication cycle and genetic reassortment: (1) schematic structure of complete influenza virion and vRNP; (2) the binding of virions to the host cell; (3) fusion between the viral envelope and the endosomal membrane. Red and blue vRNPs symbolize two distinct IAV strains during co-infection; (4) the viral genome entry to the nucleus, where replication and transcription take place; (5) genome assembly through vRNA–vRNA interactions of exposed structural motifs present in vRNPs. Compatibility of RNA packaging signals allows for co-segregation and genetic reassortment of certain segments between IAV strains. Red and blue vRNA/vRNP belong to distinct IAV strains, which undergo reassortment; and (6) accumulation of viral subunits at the budding site and the release of the new, reassortant viral progeny.

From the calculated 256 possible genetic sets which may theoretically appear during segment exchange between two strains, only a small number have been identified in natural or laboratory conditions [61,62]. Some segments have been shown to co-segregate together, leading to combinations which are identified as more frequent. The reassortment rate differs, depending on the participating strains, suggesting that a compatibility at protein or genomic level has to exist to obtain new assembly [63]. Therefore, closely related strains are more prone to undergo reassortment [64]. On the contrary, if reassortment is obstructed, the phenomenon is termed “segment mismatch” [62,65]. It has also been proven that some segment constellations cannot occur in nature, due to incompatibilities between viral and host proteins [61]. The abovementioned factors lead to reduced fitness of the progeny. However, these observations do not fully explain the course of the reassortment process, which seems to be complex and to involve factors which have not been fully described yet. Growing knowledge on RNA interactions has led to the conclusion that factors influencing genome packaging may also affect reassortment. The compatibility between vRNA packaging signals is essential for the assembly of new segment combinations in reassortant strains (Figure 2).

### 4.1. Co-Segregation of PB1 in H3N2 and H1N1 Strains

A valuable source of data comes from vaccine production procedures, where a so-called seed strain is generated through the reassortment of a seasonal influenza virus with an egg-adapted strain [66]. The main aim is to obtain a high-growth phenotype and expression of surface antigens of the seasonal isolate. Other proteins are usually derived from the egg-adapted strain, in order to assure high yield; however, some deviations have been reported. These experiments have provided information on segment incorporation preferences among strains. Analyses of reassortants generated from seasonal Udorn (H3N2) and the high-growth PR8 (H1N1) strains have revealed the preference for incorporation of the PB1 segment from the seasonal strain [66]. Interestingly, this preference was not caused by selection of the fittest virus. The abovementioned regularity was also noted in a thorough retrospective genotype investigation of over 100 reassortants originating from H3N2 and H1N1 seasonal strains, selected as a candidate for a vaccine strain [66,67,68]. This co-segregation case was carefully studied and described in the reports presented below.

Reassortation has also been studied through the use of reverse genetics. Segment exchange between the aforementioned seasonal Udorn and the high-growth PR8 was investigated in a competitive plasmid transfection models [61,67]. The results were generally consistent with the previous conclusions from vaccine production. A transfection system consisting of eight PR8 gene plasmids and an additional PB1, HA or NA coding plasmid from the Udorn strain was used [67]. These experiments demonstrated that Udorn PB1 was preferentially incorporated into the virion containing the NA gene of the same subtype. This result was confirmed by a nine-plasmid transfection model containing Udorn HA or NA, while the seven remaining segments were from PR8 and an additional PB1 segment from Udorn. On the contrary, the presence of the PR8 PB1 segment led to preferential incorporation of the PR8 NA segment. The Udorn PB1 segment was also incorporated into a majority of the produced virions in plasmid transfection containing Udorn HA and NA, as well as six remaining segments from PR8 and an additional PB1 from Udorn [61]. The co-selection was dependent on the presence of the Udorn NA segment and was not related to the replicative advantage of this particular assembly over other combinations.

Site–directed mutagenesis was applied to exchange PB1 gene fragments between strains and reveal which part of the segment is important for the incorporation into the progeny virion. The effect was studied through competitive plasmid transfection [67]. These experiments indicated the key role of the H3N2 PB1 central coding region for the co-selection of the H3N2 NA segment. Surprisingly, the terminal packaging sequences did not affect the process. In case of H1N1, it was observed that 3′ packaging sequences are relevant for the incorporation of H1N1 PB1. Further in vitro binding studies with chimeric PR8-Udorn sequences of segment PB1 and Udorn NA indicate more precisely the particular regions engaged in the interaction [61]. These tests confirmed the essential role of PB1 regions between nucleotides 1776–2070 (positive sense numbering) in the formation of PB1-NA intermolecular complexes. The sequence within this region showed complementarity to NA, making the potential vRNA interaction possible. The 1776–2070 region, although located outside previously identified packaging signals, may potentially play a role in virion packaging [61]. These findings are compliant with previous reports, which indicated that interaction loci may be located in various parts of the segments, not necessarily in the terminal regions [21,49,56].

Research carried out on reassortant viruses has indicated that the network of RNA interactions is inherited from parental strains. One of the reports focused on reassortant virus strain obtained under laboratory conditions, which acquired segments PB1 and NA from Udorn and the other vRNAs from PR8 strain (the co-segregation of vRNAs have been reported previously and described above) [9]. The strain was characterized by the presence of RNA intersegment interactions identified by the SPLASH method, which were known from both strains of origin. The differences were observed through the prevalence of certain interactions. These changes are probably necessary for the assembly of new genomic segments combinations. They are also the manifestation of the plasticity and adaptability of the influenza virus. However, these results also indicated how important certain, established interactions are for ensuring the proper course of the viral replication cycle. Interesting information derived from the experimental data indicated that one of the major interactions forms between segments PB1 and NA originating from H3N2, supporting previous observations. The previously determined segment PB1 interaction region 1776–2070 (positive sense numbering) corresponds to region 272–566 in vRNA; which includes nucleotides 305–338, identified as a prominent interaction locus with segment NA in the abovementioned SPLASH analysis.

An experiment has also been conducted concerning the reverse-engineering of viruses, in which plasmids containing vRNA segment sequences from PR8 were transfected together with plasmids containing vRNA segment sequences of other seasonal H3N2 strains [9]. The co-segregation of a seasonal H3N2 NA segment with a PB1 segment apart from Udorn was also reported for A/Memphis/1/71 (Memphis) and A/PortChalmers/1/73 (PortChalmers) strains. However, one of the tested strains did not support this regularity. Segments PB1 and NA of the A/Wyoming/3/03 (Wyoming) strain did not show any preference to assemble together. This result was consistent with that observed during the reassortation of Wyoming and PR8 in vaccine production [66]. Interestingly, the Udorn segment NA co-segregated with Wyoming segment PB1, suggesting the role of the seasonal NA vRNA sequence in the process [9]. These findings were supported by SPLASH data, showing that the sequence of identified interacting regions in segment NA are conserved in Udorn, Memphis, and PortChalmers strains, but not in Wyoming, where four single nucleotide changes are present. Further studies were conducted on Wyoming mutants, in which the conserved NA sequence was restored. Reassortment between the Wyoming mutant and PR8, and subsequent SPLASH analysis, indicated the reconstruction of the major interaction between segments PB1 and NA of H3N2 origin.

The experimental data was also consistent with the observation that avian segment PB1 and segment NA from N2 subtype tend to co-segregate in natural environments. Such an event led to the production of the Asian influenza pandemic strain in 1957 and the Hong Kong pandemic in 1968. In the first case, the (at that time) seasonal strain H1N1 acquired avian H2N2 segments PB1, HA, and NA. In the second event, the seasonal H2N2 virus reassorted with avian-origin segments PB1 and HA, through the interaction of segment NA from seasonal subtype N2 with avian segment PB1. Thorough studies on this co-segregation tendency have emphasized the influence of RNA sequence and structural changes on the fate and evolution of the virus. Further analyses of such correlations may lead to better prediction of strain reassortment, improved vaccine design, and allow for assessment of the epidemiological risks related to the production of new viral strains.

### 4.2. Other Examples of Gene Co-Selection

Another example of gene selection bias is segment HA of avian, swine or equine origin which, according to thorough analyses, is always incorporated into a human virus, together with other non-human segments [63]. The set of genes is dependent on co-infecting subtypes. In a reverse genetics approach, during exchange between Moscow (H3N2) isolated from human and avian England (H5N2), HA, PA, and M segments of avian origin segregated together [63]. Similar observations have also been made in co-infection experiments. On the contrary, in a competitive assay utilizing HA encoding plasmids from both strains and the remaining segments from H3N2, H3 subtype HA was preferentially selected. Further studies aiming to indicate specific regions responsible for co-segregation have been carried out, based on the production of chimerical segments containing sequences from H3N2 and H5N2 [63]. These experiments showed that the presence of 5′ and 3′ UTRs and terminal packaging sequences in the coding regions (80 nt at 5′ and 9 nt at 3′) identified previously by Watanabe et al. from H3N2 strain are sufficient to force the integration of the HA segment from H5N2 into the H3N2 set of segments [69]. Tests carried out on chimerical M segments allowed the authors to indicate the 3′ end of segment M H5N2 coding region as the determinant of co-segregation with segment HA H5N2 and incorporation into the H3N2 genetic background. Changes in the RNA sequence of segment M of H5N2, without altering the protein composition, influenced the genetic arrangement of progeny virions, emphasizing that interactions were present at the RNA level. The generation of single-gene reassortants proved less problematic in the case of segments presenting high sequence identity (e.g., segment NA from H5N2 into the genetic background of H3N2). The key factor for this may be the compatibility of packaging signals.

Another group of interesting processes is related to the so-called incomplete viral genomes (IVGs) present within the infected cell [58,70,71]. Reports have presented contradictory data about the abundance of these particles. However, a lack of certain segments occurs often in cells infected at low multiplicity of infection (MOI) and the frequency is strain-dependent. An association between the absence of a particular segment and reduction of the other protein expression exists [70]. In the PR8 strain, HA expression failure has been related to a reduction in NP, NA, and NS1 expression. Lack of the NP segment caused a decrease of NA and NS1 expression, while lack of the NS1 segment has been associated with a decline in NA and NP expression. Taken together, these results also support the existence of associations between vRNAs which affect protein expression and, therefore, have functional meaning. The collected experimental data concerning incomplete genome occurrence allow for the analysis of associations between segments during infection [71]. According to the results, the vRNA co-segregation of eight segments was not observed in the early stage of the viral life cycle—during replication within the cells—suggesting that these associations are not maintained throughout the viral life cycle. IVG may be reactivated by complementation in co-infection and reassortment [58,71]. In fact, the presence of incomplete genomes promotes reassortation and drives the evolution of strains.

### 4.3. Seasonal H3N2 Reassortment Potential

Reassortation events and the expansion of new viral strains among poultry has elevated the risk of avian-human IAV gene exchange [72]. A recent report has evaluated the potential compatibility of H5Nx and H7N9 subtypes with seasonal human H3N2 virus, in particular focusing on terminal RNA packaging signal regions in the HA gene, which is responsible for antigenic changes. For this purpose, chimeric viruses were generated with seasonal coding regions and terminal packaging signal regions derived from H5 or H7 HA segments. Heterologous 136 nt-long terminal packaging sequences were introduced both upstream and downstream of the open reading frames. Additionally, 20 terminal codons from both ends were silently mutated, in order to exclude their role in the process. Co-infection results showed that reassortment of the A/mute_swan/Croatia/70/2016 (H5N8) and A/Anhui/1/2013 (H7N9) into A/Panama/2007/99 (H3N2) (Panama) background was unfavorable but may occur. The further transmission of reassortant virus was also decreased suggesting the influence of incompatible packaging signals on virus fitness [72]. However, the H5 HA segment was transmitted more efficiently than H7 HA and allowed a low level of transmission in animals. It is possible that these reassortants can successfully infect new hosts in the natural environment, as there is no pre-existing immunity to the new antigenic variant. This study was consistent with previous observations carried out in H3N2 strain and H1N1 pandemic strain. An HA segment carrying Panama strain packaging sequences was preferentially packaged into the H3N2 background over HA segment carrying A/Netherlands/602/2009 (H1N1) packaging signals [65]. Interestingly, reassortment was less constrained when the exchange of packaging signals concerned NA and NS segments. This departure from the expected outcome was explained by conservation of particular (but not precisely defined) nucleotides in terminal regions or secondary structure preservation—despite sequence differences—in regions responsible for intersegment contacts. It is also possible that packaging sequences outside terminal regions, which have been revealed in other strains, allow for the maintenance of unchanged interactions of NA and NS segments with other vRNAs. The hierarchical packaging model in which certain packaging signals (in particular vRNAs) play a dominant role in virion packaging may also be meaningful, in this case. It has been previously shown that NA and NS segment packaging signals may play less important roles in genome assembly [23,73]. It should be stressed that a less stringent packaging mechanism allows a greater genetic diversification of the virus, which is critical for strain evolution.

### 4.4. Role of the NP In Virion Production

NP is a viral protein which, apart from its structural role, fulfills important functions in the replication cycle, from transcription and replication, through nuclear import and export of vRNP complexes, to genome packaging [74]. The protein regions engaged in these functions have been defined, by x-ray crystallography, as being intrinsically disordered and characterized by presence of highly conserved amino acid residues [75]. Mutations in these residues lead to replication inhibition and decreased genome packaging. Other mutational studies have revealed that particular conserved NP residues are vital for virion packaging [22,76]. The term ‘nucleoprotein code’ appeared, in the scientific literature, in the context of the NP protein contribution to the specific packaging of vRNA set [76]. The study was based on the influenza A-like viruses HL17NL10 and HL18NL11 discovered in bats, which are distantly related (their sequence identity is 50–70%, in comparison to IAV). It has been previously shown that sequence differences between IAV [SC35M (H7N7)] and IAV-like viruses lead to the incompatibility of packaging signals, preventing reassortment between them. There are also changes at the protein level, causing general protein incompatibility between IAV and IAV-like viruses. As an exception, it has been shown that IAV-like NP (HL17NL10) supported IAV polymerase activity in a polymerase reconstitution assay. However, the generation of a recombinant virus was not accomplished, even when bat NP was flanked by IAV terminal packaging sequences. The next attempt was a mutational approach, in which the conserved amino acids in IAV NP were substituted for bat HL17NL10 NP amino acids. This resulted in abnormal incorporation of vRNA to progeny virions and viral attenuation. The set of packaged segments was strictly dependent on particular amino acid exchange. Obviously, the amino acid changes originated from vRNA sequence changes, which might also have influenced the vRNA interaction loci responsible for packaging. Although the direct correlation remains elusive, it is an issue that should be investigated in the future.

## 5. Conclusions

The published data provide a consistent picture of the influenza RNA structure and interactions that influence genome assembly and progeny virion production (Figure 2). A number of conserved RNA structural motifs—mainly hairpins—have been identified within previously defined packaging signals. A subset of these have been confirmed and studied by independent researchers (Table 1). Their functional role in genome packaging has been proved through mutational studies, in which structure-disturbing mutations caused the inhibition of viral replication, segment stoichiometry changes, and defective particle production. These findings were supported by data collected from in virio experiments, showing that RNA secondary structures are formed predominantly in low-NP regions and overlap with prominent vRNA-vRNA interaction sites. Analyses of RNA regions participating in intersegment contacts have revealed a complex network which is characterized by common and strain-specific features. For reassortant viruses, in general, the interaction network is inherited from parental strains. Differences are observed in the prevalence of certain contacts and the presence of new ones. The confirmed plasticity of RNA interactions facilitates our understanding of segment incorporation into progeny virions. This complements observations made in initial experimental studies. Although the terminal regions carry packaging signals, which may be sufficient to maintain the segment contacts required for proper assembly, internal vRNA regions may also participate in the process. Studies have revealed common and unique features of RNA organization among strains. It affects segment exchange, which is less restricted in closely related strains. Some interactions seem to be inevitable for genome assembly, while others may be abolished or initiated depending on certain conditions. The presence of RNA–RNA interaction networks between segments is consistent with the co-segregation of influenza vRNAs observed during natural reassortment, lab-adaptation of strains used for vaccine production, and reverse genetics approaches. Properties which are specific to strains or lineages may serve as potential determinants of reassortment outcomes. Further studies and analyses of rules governing the assembly of new genome variants may allow researchers to predict the emergence of new strains in the future and, thus, prepare for forthcoming epidemics and pandemics.

## Figures and Tables

**Table 1:** Significant conserved RNA structural motifs with potential functional roles in IAV segment interactions and genome assembly, as supported by experimental evidence.

vRNA Segment	Nucleotide Region		Predicted Structure	Publication
	(+) Sense Numbering	(−) Sense Numbering		
1	1823–1944	398–519	motif with long helical regions and two hairpins	[41]
2	289–309	2033–2053	hairpin	[19]
	497–561	1781–1845	hairpin	[41]
5	16–39	1527–1550	hairpin	[9,25,33]
	22–68	1498–1544	two hairpins	[41]
	70–82	1484–1496	hairpin	[9,19,25]
	89–105	1461–1477	hairpin	[33]
	191–203	1363–1375	hairpin	[9,25]
	580–590	976–986	hairpin	[9,25,33]
	922–938	628–644	hairpin	[33]
	1090–1106	460–476	hairpin	[9,25]
	1144–1160	406–422	hairpin	[9,25]
	1431–1479	87–135	hairpin/pseudoknot	[9,19,25,33,41,42]
	1476–1530	36–90	hairpin	[33]
7	21–63	965–1007	dynamic	[16,26]
	219–240	788–809	hairpin	[9,26,39]
	249–260	768–779	hairpin	[9,26]
	318–333	695–710	hairpin	[9,26]
	443–450	578–585	hairpin	[9,26]
	671–691	337–357	hairpin	[9,26]
	857–890	138–171	hairpin	[9,26]
	967–994	34–61	hairpin	[9,16,26,34,36,39,43,44]
8	22–86	790–854	hairpin	[41]
	96–101/175–180	696–701/775–780	helix	[24,38]
	109–118/163–172	704–713/758–767	helix	[24,38]
	128–140	736–748	hairpin	[24,38]
	257–277	599–619	hairpin	[19]
	529–534/578–583	293–298/342–347	helix	[9,24]
	549–564	312–327	hairpin	[9,24]
	588–615	261–288	hairpin	[9,24]
